# The Role of Short-Chain Fatty Acids (SCFAs) in Colic and Anti-Inflammatory Pathways in Horses

**DOI:** 10.3390/ani15233482

**Published:** 2025-12-03

**Authors:** Nathan Schank, Ashley Cottone, Michelle Wulf, Keely Seiter, Brinley Thomas, Lynda M. J. Miller, Stacy L. Anderson, Amal Sahyoun, Ammaar H. Abidi, Modar Kassan, Ashutosh Verma

**Affiliations:** 1 Richard A. Gillespie College of Veterinary Medicine, Lincoln Memorial University, 6965 Cumberland Gap Parkway, Harrogate, TN 37752, USA; nathan.schank@lmunet.edu (N.S.); michelle.wulf@lmunet.edu (M.W.); brinley.thomas@lmunet.edu (B.T.); lynda.miller@lmunet.edu (L.M.J.M.); stacy.anderson@lmunet.edu (S.L.A.); 2 College of Dental Medicine, Lincoln Memorial University, LMU Tower, 1705 St. Mary Street, Knoxville, TN 37917, USA; ashley.cottone@lmunet.edu (A.C.); keely.seiter@lmunet.edu (K.S.); amal.sahyoun@lmunet.edu (A.S.); ammaar.abidi@lmunet.edu (A.H.A.); modar.kassan@lmunet.edu (M.K.)

**Keywords:** equine colic, short-chain fatty acids, gut microbiome, dysbiosis, gut barrier, prebiotics, probiotics

## Abstract

Equine colic remains a prevalent and potentially life-threatening condition with multifactorial origins, including dietary imbalances, stress, and microbial dysbiosis. Central to equine gut health is the production of short-chain fatty acids (SCFAs), primarily acetate, propionate, and butyrate, generated through microbial fermentation of dietary fibers in the hindgut. These metabolites not only serve as vital energy sources but also play crucial roles in maintaining intestinal barrier integrity, modulating motility, and suppressing inflammation. This review explores the role of SCFAs in equine gastrointestinal health, with particular emphasis on their anti-inflammatory effects and potential to prevent or mitigate colic. We examine how SCFAs interact with immune pathways, via G-protein-coupled receptors and regulatory T-cell promotion, to reduce pro-inflammatory cytokines such as TNF-α and IL-6. Evidence suggests that dietary shifts toward high-starch or low-fiber intake can reduce SCFA production, contributing to microbial imbalance, increased gut permeability, and systemic inflammation, all hallmarks of colic pathophysiology. Strategies to enhance SCFA levels, including high-forage diets, targeted prebiotic and probiotic supplementation, and emerging approaches like fecal microbiota transplantation, are discussed. Despite promising findings, significant gaps remain in equine-specific research, highlighting the need for longitudinal and mechanistic studies. Understanding and harnessing the therapeutic potential of SCFAs could pave the way for novel, microbiome-based interventions in colic prevention and treatment.

## Simple Summary

Colic is a serious condition in horses, often initiated by factors such as diet changes, stress, and imbalances in good bacteria in the gut. A healthy equine gut relies on a byproduct of good bacteria, called short-chain fatty acids (SCFAs), produced when gut bacteria ferment fiber in the hindgut. These SCFAs provide energy for the horse and help keep lining of the gut strong, control gut movement, and reduce inflammation. When horses eat too much starch or too little fiber, SCFA levels can drop, leading to gut imbalance, leaky intestines, and inflammation, all of which increase the risk of colic. This review highlights how SCFAs influence gut and immune health, especially by lowering harmful inflammatory molecules in the body. It also explores strategies to naturally boost SCFA production, such as feeding high-forage diets, adding prebiotics and probiotics, and using new methods like fecal transplantation. While early research is promising, more long-term studies in horses are needed. Understanding how SCFAs support gut health could lead to new ways to prevent or manage colic.

## 1. Introduction

Gastrointestinal health is fundamental to the well-being of horses, which requires an optimal microbiome for the efficient fermentation of dietary fiber and energy production [1]. An increasing number of studies highlight the central role of the equine gut microbiome in maintaining gastrointestinal homeostasis, with its disruption leading to dysbiosis, impaired fermentation, inflammation, and increased risk of colic [2,3,4,5]. Colic, a broad term used to describe abdominal pain, represents one of the leading causes of morbidity and mortality in horses and remains among the most common conditions necessitating emergency intervention [6,7].

Colic often results from impaired ingesta flow, which may arise from either physical or functional obstructions [8]. More specific categories include gas accumulation, impactions, intestinal displacement, torsions, lipomas, and entrapments [6]. There is considerable variability in the proportion of colic cases requiring medical (50.3–96%) versus surgical (4–49.7%) intervention, influenced by factors such as chronicity, geographic region, age, underlying etiology, and other variables [9,10,11,12,13,14]. Reported incidence range from 4.2 to 10.6 events per 100 horses, with estimated annual treatment costs reaching $115 million in the United States [15]. Clinical presentation is often nonspecific, manifesting as abdominal kicking, inappetence, rolling, or pawing, making definitive diagnosis difficult without advanced diagnostic tools. Rectal palpation, however, can help identify the affected gastrointestinal segment(s). Breed and use may also influence susceptibility; for example, colic occurs more frequently in Thoroughbreds, whereas Arabians tends to exhibit lower incidence rates [16]. Horses in training or used for eventing demonstrate a higher colic risk than non-active horses [17], potentially due to factors such as higher-intensity training schedules, frequent changes in management or feeding practices, stress associated with travel and competition, and increased exposure to environmental or physiological stressors. Sudden dietary changes, reduced forage intake, high concentrate feeding, use of nonsteroidal anti-inflammatory drugs (NSAIDs), and various management-related stressors have been consistently identified as major risk factors for colic [18,19,20,21]. Additionally, enteritis and colitis represent important inflammatory contributors to colic syndromes, often complicating diagnosis and management [22].

As hindgut fermenters, horses rely on microbial activity in the cecum, large colon, and small colon for the digestion of plant materials [23]. Unlike ruminants, horses obtain most of their energy through microbial degradation of complex carbohydrates in the hindgut [24]. The equine hindgut microbiome, comprising bacteria, yeasts, fungi, and protozoa, facilitates the breakdown of cellulose, hemicellulose, and pectin into short-chain fatty acids (SCFAs), which serve as a critical energy source [25,26]. Without this microbial ecosystem, horses would lack the enzymatic capacity necessary to digest dietary fibers efficiently.

SCFAs, defined as fatty acids containing fewer than six carbon atoms, exert diverse metabolic and protective effects both locally within the hindgut and systemically throughout the host [27]. The principal SCFAs produced in the equine hindgut, acetate, propionate, and butyrate, not only provide an essential energy substrate for the horse but also support the proliferation of fibrolytic microbes and contribute to intestinal health modulation [28].

Although alterations in SCFA production have been associated with gastrointestinal dysbiosis and colic, the direct contribution of SCFAs to the pathophysiology of colic remains poorly understood. The objective of this review is to critically evaluate the current literature on the role of SCFAs in equine health with particular emphasis on their involvement in colic and inflammation. In addition, the review outlines mechanisms by which SCFAs affect gastrointestinal health, identify potential areas of further investigation, and inform strategies to improve equine health, mitigate colic risks and reduce inflammation.

## 2. Short-Chain Fatty Acids: Production and Functions

The majority of SCFAs in the equine hindgut are produced by resident bacterial populations through the fermentation of dietary fibers. The dominant bacterial phyla include Firmicutes and Bacteroidetes, along with clusters of Clostridium species. Within Firmicutes, members of the Lachnospiraceae and Ruminococcaceae families play a key role in SCFA production [29,30]. Members of the genus Fibrobacter are also abundant in the hindgut and contribute significantly to cellulose degradation [31].

Dietary composition strongly influences microbial composition and, consequently, SCFA production. Arnold et al. (2021) demonstrated that diet significantly alters the equine fecal microbiome, with notable shifts in bacterial populations across forage-only and forage-plus-concentrate diets [32]. High-forage diets support a diverse and abundant fibrolytic microbial community, whereas starch-rich diets, even when fed in moderate amounts, promote lactate-producing bacteria at the expense of SCFA-producing species [33].

Among the SCFAs, butyrate is particularly important, serving as the primary energy source for colonocytes and playing a critical role in maintaining intestinal barrier integrity. Butyrate regulates epithelial tight junctions, thereby reducing translocation of pathogenic bacteria into the bloodstream, and promotes intestinal mucus secretion as an additional protective mechanism. SCFAs also activate G-protein-coupled receptors (GPCRs), stimulating the release of peptide YY and glucagon-like peptide-1 (GLP-1), and interact with other GPCRs involved in glucose and lipid metabolism [34,35]. Because of their small molecular structure, SCFAs can cross the blood–brain barrier, where they induce neurotransmitter secretion of both γ-aminobutyric acid (GABA) and serotonin, as well as promote neuronal growth and decrease neuroinflammation [34,36].

Beyond their metabolic functions, SCFAs exert significant effects on intestinal immunity. They enhance mucus production, stimulate secretion of antimicrobial peptides, and regulate inflammation. SCFAs are ligands for GPCRs, particularly GPR-41 and GPR-43, which are expressed on intestinal epithelial cells and resident leukocytes, and influence immune signaling [37,38,39,40]. SCFAs further modulate T-cell differentiation, promoting the generation of regulatory T-cells and the production of the anti-inflammatory cytokine interleukin-10 (IL-10). Collectively, these mechanisms regulate neutrophil, monocyte, and macrophage activity, contributing to the maintenance of intestinal immune homeostasis [35,36,41,42].

SCFAs also play broader roles in systemic immunity and disease modulation. Their effects can be pro- or anti-inflammatory, depending on concentration and context. For example, at low concentrations, butyrate serves as an energy source for both healthy and tumor cells; However, at high concentrations it induces cell-cycle arrest, apoptosis, and the expression of anti-metastatic genes [34].

## 3. SCFA Deficiency and Its Association with Colic

Alterations in the gastrointestinal microbiome are well-documented during periods of disease. A recent study of a feral horse population on Sable Island, Nova Scotia, showed that horses with higher relative abundance of Fibrobacter succinogenes, a fibrolytic bacterium whose primary metabolic byproducts are the SCFAs acetate and succinate, had improved survival, while those with reduced survivability harbored higher levels of methane-producing bacteria [43]. These findings highlight the role of gastrointestinal microbial balance in equine health, maintained through a complex interplay of factors, as reviewed by Chaucheyras-Durand et al., 2022 [44].

SCFAs exert potent anti-inflammatory effects by reducing chemokine production and limiting leukocyte recruitment. GPR-41, expressed on neutrophils, monocytes, and adipocytes, is activated by SCFAs and can induce neutrophilic chemotaxis. However, in the presence of other chemoattractants, SCFAs exert the opposite effect by downregulating receptor expression, thereby suppressing neutrophil migration. Butyrate further contributes by inhibiting T-cell proliferation, reducing antigen-driven T-lymphocyte expansion, and promoting regulatory T-cell differentiation which collectively suppress excessive immune activation [27,45,46,47].

Several studies have examined the association between diet-induced dysbiosis, alterations in SCFAs and occurrence of colic. Stewart et al. (2019) [48] demonstrated that horses with higher Firmicutes-to-Proteobacteria ratios had a lower incidence of colic, whereas postpartum mares with colic showed increased abundance of Proteobacteria [3,48]. Across multiple studies, colic horses exhibit reduced microbial diversity, characterized by decreases in Firmicutes and Bacteroidetes and concomitant increases in Proteobacteria [5]. Dysbiosis involving increased methane-producing bacteria, loss of fibrolytic taxa, and enrichment of Proteobacteria has been linked to higher lactate production, reduced hindgut pH, diminished fibrolytic activity, and ultimately decreased SCFA output [44].

SCFAs, particularly butyrate, are critical for maintaining epithelial integrity and reducing inflammation both locally and systemically. In inflamed intestinal states, increased permeability combined with reduced SCFA production heightens the risk of endotoxemia [48]. SCFAs promote neutrophil apoptosis, restore barrier integrity, and modulate Toll-like receptor signaling [27,47]. Given that equine colitis is characterized by delayed neutrophil apoptosis and dysregulated inflammation [49], SCFA supplementation or modulation may represent a promising therapeutic avenue for restoring mucosal homeostasis.

**Figure 1:**
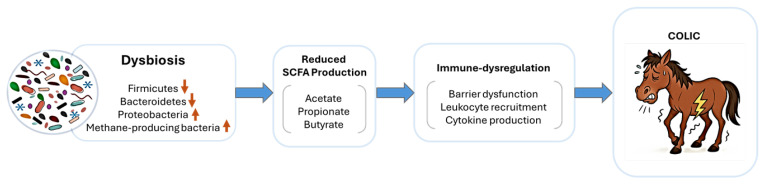
**Proposed mechanism linking microbial dysbiosis, short-chain fatty acid (SCFA) depletion, and immune dysfunction in equine gut health**. Gut dysbiosis reduces populations of fibrolytic and butyrate-producing bacteria, leading to diminished SCFA synthesis. Reduced SCFA availability impairs epithelial cell metabolism, weakens tight junction integrity, and heightens intestinal permeability. The resulting barrier dysfunction facilitates translocation of endotoxins, triggering systemic inflammation and immune dysregulation. Collectively, these interrelated processes perpetuate a cycle of inflammation and epithelial damage that underlies colic, and possibly other gastrointestinal pathologies in horses.

Horses with prolonged hindgut acidosis, often triggered by sudden dietary changes and characterized by a decline in fibrolytic bacteria and reduced SCFA production, face an increased risk of colic [23] (Figure 1). Laminitis and hindgut acidosis models induced with oligofructose provide further evidence of disrupted microbial and metabolomic profiles, including reduced SCFA levels [50]. Observational studies have consistently demonstrated altered SCFA profiles in colic horses, and experimental approaches such as butyrate infusion in other host species have shown therapeutic promise. Future investigations should prioritize longitudinal assessment of SCFA levels before, during and after colic events to better understand whether SCFA depletion represents a causal predisposing factor or a secondary consequence of the disease process.

## 4. Strategies to Enhance SCFA Production for Colic Prevention

**Figure 2:**
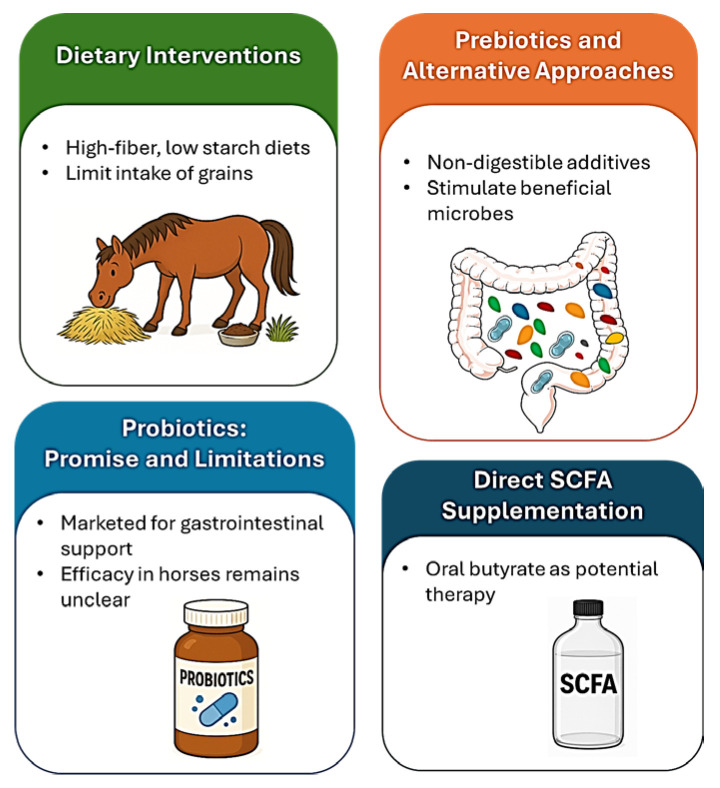
**Schematic overview of dietary and microbiota-based strategies to enhance short-chain fatty acid (SCFA) production for colic prevention**. High-fiber, low-starch diets support fibrolytic bacteria and sustained SCFA synthesis, while probiotics and prebiotics such as inulin, fructooligosaccharides, and mannan-oligosaccharides enrich beneficial taxa. Increased acetate, propionate, and butyrate strengthen epithelial junctions, modulate immune responses, and reduce mucosal inflammation. Translational studies further support SCFA-centered interventions, including direct butyrate supplementation, as promising approaches to improve gut barrier integrity and decrease colic susceptibility in horses.

Diet, microbiota-targeted interventions, and direct butyrate supplementation offer promising avenues to enhance short-chain fatty acid (SCFA) production and maintain gastrointestinal health in horses (Figure 2). These strategies are discussed in this section.

### 4.1. Dietary Interventions

Diet is a central determinant of gastrointestinal stability. Durham (2009) demonstrated that slow, continuous consumption of high-fiber, low-starch diets best support fermentative fibrolytic bacteria and promotes intestinal homeostasis [51]. Conversely, intermittent feeding or abrupt replacement of major feed components disrupts microbial populations and increases the risk of gastrointestinal disturbance. Similarly, Blikslager (2019) reported that high-starch diets, compared with forage-only diets, promote lactic acid-producing bacteria, predisposing horses to colonic distension and impaction [52]. These findings emphasize the importance of dietary regimens tailored to both the nutritional needs of the horse and the potential gastrointestinal risks of each feeding strategy.

High-fiber diets provide a stable substrate for hindgut fermentation and are associated with greater SCFA production [53]. In contrast, starch-heavy diets shift microbial populations toward lactate producers, increasing the risk of laminitis and colic. Morrison et al. (2020) noted that horses fed hay-only diets exhibited higher acetate concentrations than those fed grain and hay [54]. Current feeding guidelines recommend limiting starch to ≤2 g/kg bodyweight per meal while ensuring daily forage intake of 1.5–2% of body weight as long-stem fiber. Inclusion of fermentable fiber sources such as beet pulp, soy hulls, and alfalfa can further promote hindgut fermentation. Horses with continuous access to hay demonstrate a reduced incidence of colic compared to those fed once or twice daily [55,56].

### 4.2. Probiotics: Promise and Limitations

Probiotics are widely marketed for equine gastrointestinal support; however, evidence supporting their efficacy in equine gastrointestinal disease remains limited [57]. Weese (2002) reported that only 2 of 13 examined equine and human probiotic products accurately reflected their contents [58]. Subsequent reviews have confirmed frequent discrepancies in organism viability, concentration, and even presence [59]. Factors such as improper storage by owners or inadequate manufacturer guidance further compromise product integrity.

While probiotics hold theoretical potential to improve gastrointestinal health and aid recovery post-disease, issues related to quality control issues, lack of standardized strains, and insufficient in vivo data hinder their clinical application [60,61]. Recent reviews also highlight the complexity of the equine hindgut microbiome and the challenges this poses for consistent probiotic efficacy [62].

### 4.3. Prebiotics and Alternative Approaches

Prebiotics are non-digestible dietary additives that selectively stimulate beneficial microbes and have gained increasing attention as adjuncts to enhance SCFA production. They are typically composed of complex carbohydrates such as fructo-oligosaccharides (FOS), galacto-oligosaccharides (GOS), inulin, and resistant starches. These compounds resist enzymatic digestion in the upper gastrointestinal tract and undergo fermentation by hindgut microbiota [63]. The resulting metabolites, particularly SCFAs, play key roles in maintaining gut health, modulating immunity, and improving nutrient absorption. The concept of prebiotics has been widely applied in both human and veterinary nutrition to enhance gastrointestinal health and confer systemic benefits through microbiome modulation [63].

Various prebiotics have been investigated in animals, including mannan-oligosaccharides (MOS), FOS, GOS, and inulin, each differing in fermentability and effects on microbial communities [64,65]. For instance, FOS and inulin have consistently been shown to enrich populations of beneficial Bifidobacteria and Lactobacilli in pigs and poultry [66]. In horses, the large cecum and colon provide an ideal environment for fiber fermentation, making them particularly responsive to prebiotic supplementation. Experimental studies demonstrate that inulin and FOS can mitigate gastrointestinal disturbances and enhance microbial stability in equines [67,68].

Controlled trials indicate that FOS supplementation can increase fibrolytic bacterial populations and stimulate SCFA production, thereby promoting colonic health and improving feed efficiency [69]. Beyond digestive benefits, prebiotics may also support immune regulation, limit pathogen colonization, and preserve gut barrier integrity in horses and other livestock [67]. In equine practice, prebiotic and symbiotic (pre-and probiotic combined) supplementation has shown promise in improving fecal microbial profiles, reducing colic risk, and supporting gut function during stressors such as transportation or abrupt dietary changes [68,70,71,72,73]. However, because of the wide variety of prebiotic compounds and probiotic strains used across studies, further research is needed to draw more definitive conclusions. 

### 4.4. Direct SCFA Supplementation

In human patients with inflammatory bowel disease, diets promoting SCFA production improved clinical outcomes, epithelial proliferation, and microbial balance [74]. Rodent models have further demonstrated reduced inflammation, improved cytokine profiles, and enhanced mucosal healing with SCFA-targeted interventions [27,47,75,76].

Given the central role of SCFAs in gastrointestinal health, direct SCFA supplementation represents a logical future direction. Oral butyrate supplementation is already available for human use, and SCFA derivatives are being actively investigated in human clinical studies [77]. Translating these strategies to equine medicine will require rigorous trials to evaluate formulation, delivery, dosing, and safety. Nonetheless, the growing body of evidence supports SCFA modulation, either through dietary, prebiotic, or direct supplementation, as a promising therapeutic frontier for improving gut health and preventing colic in horses.

## 5. Future Research and Therapeutic Potential

Despite increasing recognition of the critical role of short-chain fatty acids (SCFAs) in equine gastrointestinal health, there remains a paucity of equine-specific studies. Much of the current understanding of SCFA production, utilization, and function derives from human and murine models [37]. While these insights are valuable, equine-specific investigations are needed to clarify species-level differences in microbial metabolism and host–microbe interactions. Longitudinal cohort studies that follow horses across their lifetimes, correlating diet, microbiome dynamics, SCFA profiles, and disease incidence, would provide valuable data to strengthen causal associations, provide information on any species-specific adverse effects, and inform evidence-based preventative strategies.

Fecal microbiota transplantation (FMT) represents another promising yet underexplored area. Widely used in ruminants to treat dysbiosis, and increasingly studied in other species [78], FMT could hold therapeutic potential in equine medicine. However, equine-specific protocols remain undeveloped. Currently, there is no established dysbiosis index for horses, and the efficacy and safety of FMT in this species remain to be determined [26]. Developing standardized methodologies, including donor screening, microbial characterization, and monitoring outcomes, will be essential steps before FMT can be integrated into equine practice.

Direct SCFA supplementation represents a logical extension of current microbiome-based strategies. Given the established roles of SCFAs in maintaining barrier integrity, immune modulation, and colic prevention, targeted supplementation may offer significant therapeutics benefits for equine health and welfare. Nonetheless, several challenges must be addressed. Effective approaches would likely require strain-specific probiotic formulations capable of colonizing the hindgut, or the development of stable SCFA derivatives that can withstand foregut digestion and be delivered intact to the large intestine. Current progress in other species may offer translational guidance. Oral butyrate supplementation is already available for humans, and in vitro studies in swine have demonstrated promising anti-inflammatory and anti-diarrheal effects [77]. However, to date, no comparable studies have been conducted in horses.

Advancing equine-specific SCFA research thus represents a critical next step toward leveraging the therapeutic potential of SCFAs in preventing colic, mitigating gastrointestinal disease, and improving equine health outcomes.

## 6. Conclusions

SCFAs are fundamental to equine gastrointestinal health, where they act locally to preserve epithelial barrier function and systemically to support energy metabolism, immune modulation, and cellular signaling. Among these metabolites, butyrate exerts potent anti-inflammatory effects by regulating immune responses, maintaining barrier integrity, and suppressing pro-inflammatory pathways. Collectively, these protective functions highlight the therapeutic potential of SCFAs in the prevention and management of gastrointestinal disorders, including colic.

Despite their importance, equine-specific research on SCFAs remains limited, with much of the current knowledge extrapolated from human and rodent studies. Advancing this field will require longitudinal and controlled studies in horses to clarify the relationships among diet, microbiome composition, SCFA dynamics, and disease outcomes. Future research should also prioritize the development of nutritional and microbial strategies that enhance SCFA production in the hindgut, as well as rigorous evaluations of SCFA supplementation approaches.

Bridging these knowledge gaps will enable the equine veterinary community to translate SCFA-centered findings into evidence-based management practices. Such advancements hold substantial promises for reducing the incidence of colic, strengthening gastrointestinal resilience, and ultimately improving equine health, performance, and welfare.

## Data Availability

No new data were created or analyzed in this study. Data sharing is not applicable.

## Abbreviations

The following abbreviations are used in this manuscript:

SCFA	short-chain fatty acids
GPCR	G-protein-coupled receptors
GLP-1	glucagon-like peptide-1
FMT	Fecal microbiota transplantation
GABA	γ-aminobutyric acid
FOS	fructo-oligosaccharides
GOS	galacto-oligosaccharides

## References

[1] Venable E.B., Bland S.D., McPherson J.L., Francis J. (2016). Role of the gut microbiota in equine health and disease. Anim. Front..

[2] Salem S.E., Maddox T.W., Berg A., Antczak P., Ketley J.M., Williams N.J., Archer D.C. (2018). Variation in faecal microbiota in a group of horses managed at pasture over a 12-month period. Sci. Rep..

[3] Weese J.S., Holcombe S.J., Embertson R.M., Kurtz K.A., Roessner H.A., Jalali M., Wismer S.E. (2015). Changes in the faecal microbiota of mares precede the development of post partum colic. Equine Vet. J..

[4] Arnold C.E., Pilla R. (2023). What Is the Microbiota and What Is Its Role in Colic?. Vet. Clin. N. Am. Equine Pract..

[5] Lara F., Castro R., Thomson P. (2022). Changes in the gut microbiome and colic in horses: Are they causes or consequences?. Open Vet. J..

[6] Reed S.M., Bayly W.M., Sellon D.C. (2017). Equine Internal Medicine.

[7] Eighner K. Colic survival rate: Review of 254 Cases. 19 March 2020. https://vetmed.illinois.edu/2020/03/19/colic-survival-rate/.

[8] Orsini J.A., Divers T.J. (2013). Equine Emergencies: Treatment and Procedures.

[9] Singer E.R., Smith M.A. (2002). Examination of the horse with colic: Is it medical or surgical?. Equine Vet. Educ..

[10] Proudman C.J., Smith J.E., Edwards G.B., French N.P. (2002). Long-term survival of equine surgical colic cases. Part 1: Patterns of mortality and morbidity. Equine Vet. J..

[11] Proudman C.J. (1992). A two year, prospective survey of equine colic in general practice. Equine Vet. J..

[12] Tinker M.K., White N.A., Lessard P., Thatcher C.D., Pelzer K.D., Davis B., Carmel D.K. (1997). Prospective study of equine colic incidence and mortality. Equine Vet. J..

[13] Abutarbush S.M., Carmalt J.L., Shoemaker R.W. (2005). Causes of gastrointestinal colic in horses in western Canada: 604 cases (1992 to 2002). Can. Vet. J..

[14] Hillyer M.H., Taylor F.G., French N.P. (2001). A cross-sectional study of colic in horses on thoroughbred training premises in the British Isles in 1997. Equine Vet. J..

[15] Traub-Dargatz J.L., Kopral C.A., Seitzinger A.H., Garber L.P., Forde K., White N.A. (2001). Estimate of the national incidence of and operation-level risk factors for colic among horses in the United States, spring 1998 to spring 1999. J. Am. Vet. Med. Assoc..

[16] United States Department of Agriculture, National Animal Health Monitoring System (1998). Part I: Baseline Reference of 1998 Equine Health and Management.

[17] Cohen N.D., Peloso J.G. (1996). Risk factors for history of previous colic and for chronic, intermittent colic in a population of horses. J. Am. Vet. Med. Assoc..

[18] Matyjaszek S.A., Morton A.J., Freeman D.E., Grosche A., Polyak M.M., Kuck H. (2009). Effects of flunixin meglumine on recovery of colonic mucosa from ischemia in horses. Am. J. Vet. Res..

[19] Marshall J.F., Blikslager A.T. (2011). The effect of nonsteroidal anti-inflammatory drugs on the equine intestine. Equine Vet. J. Suppl..

[20] Whitfield-Cargile C.M., Coleman M.C., Cohen N.D., Chamoun-Emanuelli A.M., DeSolis C.N., Tetrault T., Sowinski R., Bradbery A., Much M. (2021). Effects of phenylbutazone alone or in combination with a nutritional therapeutic on gastric ulcers, intestinal permeability, and fecal microbiota in horses. J. Vet. Intern. Med..

[21] Fernandes K.A., Kittelmann S., Rogers C.W., Gee E.K., Bolwell C.F., Bermingham E.N., Thomas D.G. (2014). Faecal microbiota of forage-fed horses in New Zealand and the population dynamics of microbial communities following dietary change. PLoS ONE.

[22] Feary D.J., Hassel D.M. (2006). Enteritis and colitis in horses. Vet. Clin. N. Am. Equine Pract..

[23] Dicks L., Botha M., Dicks E., Botes M. (2014). The equine gastro-intestinal tract: An overview of the microbiota, disease and treatment. Livest. Sci..

[24] Geor R.J., Harris P.A., Coenen M. (2013). Equine Applied and Clinical Nutrition: Health, Welfare and Performance.

[25] von Engelhardt W., Bartels J., Kirschberger S., Meyer zu Düttingdorf H.D., Busche R. (1998). Role of short-chain fatty acids in the hind gut. Vet. Q..

[26] Boucher L., Leduc L., Leclere M., Costa M.C. (2024). Current Understanding of Equine Gut Dysbiosis and Microbiota Manipulation Techniques: Comparison with Current Knowledge in Other Species. Animals.

[27] Vinolo M.A., Rodrigues H.G., Nachbar R.T., Curi R. (2011). Regulation of inflammation by short chain fatty acids. Nutrients.

[28] Julliand V., Grimm P. (2017). The Impact of Diet on the Hindgut Microbiome. J. Equine Vet. Sci..

[29] Garber A., Hastie P., Murray J.A. (2020). Factors Influencing Equine Gut Microbiota: Current Knowledge. J. Equine Vet. Sci..

[30] Kauter A., Epping L., Semmler T., Antao E.M., Kannapin D., Stoeckle S.D., Gehlen H., Lubke-Becker A., Gunther S., Wieler L.H. (2019). The gut microbiome of horses: Current research on equine enteral microbiota and future perspectives. Anim. Microbiome.

[31] Neumann A.P., McCormick C.A., Suen G. (2017). Fibrobacter communities in the gastrointestinal tracts of diverse hindgut-fermenting herbivores are distinct from those of the rumen. Environ. Microbiol..

[32] Arnold C.E., Pilla R., Chaffin M.K., Leatherwood J.L., Wickersham T.A., Callaway T.R., Lawhon S.D., Lidbury J.A., Steiner J.M., Suchodolski J.S. (2021). The effects of signalment, diet, geographic location, season, and colitis associated with antimicrobial use or Salmonella infection on the fecal microbiome of horses. J. Vet. Intern. Med..

[33] Muhonen S., Sadet-Bourgeteau S., Julliand V. (2021). Effects of Differences in Fibre Composition and Maturity of Forage-Based Diets on the Microbial Ecosystem and Its Activity in Equine Caecum and Colon Digesta and Faeces. Animals.

[34] Lange O., Proczko-Stepaniak M., Mika A. (2023). Short-Chain Fatty Acids-A Product of the Microbiome and Its Participation in Two-Way Communication on the Microbiome-Host Mammal Line. Curr. Obes. Rep..

[35] Kimura I., Ozawa K., Inoue D., Imamura T., Kimura K., Maeda T., Terasawa K., Kashihara D., Hirano K., Tani T. (2013). The gut microbiota suppresses insulin-mediated fat accumulation via the short-chain fatty acid receptor GPR43. Nat. Commun..

[36] Koh A., De Vadder F., Kovatcheva-Datchary P., Backhed F. (2016). From Dietary Fiber to Host Physiology: Short-Chain Fatty Acids as Key Bacterial Metabolites. Cell.

[37] Kimura I., Ichimura A., Ohue-Kitano R., Igarashi M. (2020). Free Fatty Acid Receptors in Health and Disease. Physiol. Rev..

[38] Le Poul E., Loison C., Struyf S., Springael J.Y., Lannoy V., Decobecq M.E., Brezillon S., Dupriez V., Vassart G., Van Damme J. (2003). Functional characterization of human receptors for short chain fatty acids and their role in polymorphonuclear cell activation. J. Biol. Chem..

[39] Nilsson N.E., Kotarsky K., Owman C., Olde B. (2003). Identification of a free fatty acid receptor, FFA2R, expressed on leukocytes and activated by short-chain fatty acids. Biochem. Biophys. Res. Commun..

[40] Brown A.J., Goldsworthy S.M., Barnes A.A., Eilert M.M., Tcheang L., Daniels D., Muir A.I., Wigglesworth M.J., Kinghorn I., Fraser N.J. (2003). The Orphan G protein-coupled receptors GPR41 and GPR43 are activated by propionate and other short chain carboxylic acids. J. Biol. Chem..

[41] Maslowski K.M., Vieira A.T., Ng A., Kranich J., Sierro F., Yu D., Schilter H.C., Rolph M.S., Mackay F., Artis D. (2009). Regulation of inflammatory responses by gut microbiota and chemoattractant receptor GPR43. Nature.

[42] Singh N., Gurav A., Sivaprakasam S., Brady E., Padia R., Shi H., Thangaraju M., Prasad P.D., Manicassamy S., Munn D.H. (2014). Activation of Gpr109a, receptor for niacin and the commensal metabolite butyrate, suppresses colonic inflammation and carcinogenesis. Immunity.

[43] Stothart M.R., McLoughlin P.D., Medill S.A., Greuel R.J., Wilson A.J., Poissant J. (2024). Methanogenic patterns in the gut microbiome are associated with survival in a population of feral horses. Nat. Commun..

[44] Chaucheyras-Durand F., Sacy A., Karges K., Apper E. (2022). Gastro-Intestinal Microbiota in Equines and Its Role in Health and Disease: The Black Box Opens. Microorganisms.

[45] Tan J., McKenzie C., Potamitis M., Thorburn A.N., Mackay C.R., Macia L. (2014). The role of short-chain fatty acids in health and disease. Adv. Immunol..

[46] Li M., van Esch B., Wagenaar G.T.M., Garssen J., Folkerts G., Henricks P.A.J. (2018). Pro- and anti-inflammatory effects of short chain fatty acids on immune and endothelial cells. Eur. J. Pharmacol..

[47] Correa-Oliveira R., Fachi J.L., Vieira A., Sato F.T., Vinolo M.A. (2016). Regulation of immune cell function by short-chain fatty acids. Clin. Transl. Immunol..

[48] Stewart H.L., Southwood L.L., Indugu N., Vecchiarelli B., Engiles J.B., Pitta D. (2019). Differences in the equine faecal microbiota between horses presenting to a tertiary referral hospital for colic compared with an elective surgical procedure. Equine Vet. J..

[49] Anderson S.L., Singh B. (2017). Neutrophil apoptosis is delayed in an equine model of colitis: Implications for the development of systemic inflammatory response syndrome. Equine Vet. J..

[50] Tuniyazi M., He J., Guo J., Li S., Zhang N., Hu X., Fu Y. (2021). Changes of microbial and metabolome of the equine hindgut during oligofructose-induced laminitis. BMC Vet. Res..

[51] Durham A.E. (2009). The role of nutrition in colic. Vet. Clin. N. Am. Equine Pract..

[52] Blikslager A.T. (2019). Colic Prevention to Avoid Colic Surgery: A Surgeon’s Perspective. J. Equine Vet. Sci..

[53] Santos A.S., Rodrigues M.A., Bessa R.J., Ferreira L.M., Martin-Rosset W. (2011). Understanding the equine cecum-colon ecosystem: Current knowledge and future perspectives. Animal.

[54] Morrison P.K., Newbold C.J., Jones E., Worgan H.J., Grove-White D.H., Dugdale A.H., Barfoot C., Harris P.A., Argo C.M. (2020). The equine gastrointestinal microbiome: Impacts of weight-loss. BMC Vet. Res..

[55] Harris P.A., Ellis A.D., Fradinho M.J., Jansson A., Julliand V., Luthersson N., Santos A.S., Vervuert I. (2017). Review: Feeding conserved forage to horses: Recent advances and recommendations. Animal.

[56] Cipriano-Salazar M., Adegbeye M.J., Elghandour M.M., Barbabosa-Pilego A., Mellado M., Hassan A., Salem A.Z.M. (2019). The Dietary Components and Feeding Management as Options to Offset Digestive Disturbances in Horses. J. Equine Vet. Sci..

[57] Schoster A. (2018). Probiotic Use in Equine Gastrointestinal Disease. Vet. Clin. N. Am. Equine Pract..

[58] Weese J.S. (2002). Microbiologic evaluation of commercial probiotics. J. Am. Vet. Med. Assoc..

[59] Berreta A., Burbick C.R., Alexander T., Kogan C., Kopper J.J. (2021). Microbial Variability of Commercial Equine Probiotics. J. Equine Vet. Sci..

[60] Berreta A., Kopper J. (2022). Equine Probiotics-What Are They, Where Are We and Where Do We Need To Go?. J. Equine Vet. Sci..

[61] Schoster A., Weese J.S., Guardabassi L. (2014). Probiotic use in horses—What is the evidence for their clinical efficacy?. J. Vet. Intern. Med..

[62] Weinert-Nelson J., Williams C. (2023). The Equine Hindgut Microbiome. Rutgers NJAES Fact Sheet. https://njaes.rutgers.edu/e375/.

[63] Gibson G.R., Hutkins R., Sanders M.E., Prescott S.L., Reimer R.A., Salminen S.J., Scott K., Stanton C., Swanson K.S., Cani P.D. (2017). Expert consensus document: The International Scientific Association for Probiotics and Prebiotics (ISAPP) consensus statement on the definition and scope of prebiotics. Nat. Rev. Gastroenterol. Hepatol..

[64] Bachmann M., Glatter M., Bochnia M., Greef J.M., Breves G., Zeyner A. (2021). Degradation of Monosaccharides, Disaccharides, and Fructans in the Stomach of Horses Adapted to a Prebiotic Dose of Fructooligosaccharides and Inulin. J. Equine Vet. Sci..

[65] Bachmann M., Glatter M., Bochnia M., Wensch-Dorendorf M., Greef J.M., Breves G., Zeyner A. (2020). In Vitro Gas Production from Batch Cultures of Stomach and Hindgut Digesta of Horses Adapted to a Prebiotic Dose of Fructooligosaccharides and Inulin. J. Equine Vet. Sci..

[66] Gaggia F., Mattarelli P., Biavati B. (2010). Probiotics and prebiotics in animal feeding for safe food production. Int. J. Food Microbiol..

[67] Respondek F., Goachet A.G., Julliand V. (2008). Effects of dietary short-chain fructooligosaccharides on the intestinal microflora of horses subjected to a sudden change in diet. J. Anim. Sci..

[68] Glatter M., Borewicz K., van den Bogert B., Wensch-Dorendorf M., Bochnia M., Greef J.M., Bachmann M., Smidt H., Breves G., Zeyner A. (2019). Modification of the equine gastrointestinal microbiota by Jerusalem artichoke meal supplementation. PLoS ONE.

[69] Liu S., Chen Y., Li J., Yang M. (2025). Editorial: Unlocking the power of gut microbiota to improving health and welfare in non-ruminant livestock. Front. Vet. Sci..

[70] Ford T., McAdams Z.L., Townsend K.S., Martin L.M., Johnson P.J., Ericsson A.C. (2023). Effect of Sugar Beet Pulp on the Composition and Predicted Function of Equine Fecal Microbiota. Biology.

[71] McGilloway M., Manley S., Aho A., Heeringa K.N., Whitacre L., Lou Y., Squires E.J., Pearson W. (2023). Dietary Fermentation Product of Aspergillus Oryzae Prevents Increases in Gastrointestinal Permeability (‘Leaky Gut’) in Horses Undergoing Combined Transport and Exercise. Animals.

[72] MacNicol J.L., Renwick S., Ganobis C.M., Allen-Vercoe E., Weese J.S., Pearson W. (2023). The influence of a probiotic/prebiotic supplement on microbial and metabolic parameters of equine cecal fluid or fecal slurry in vitro. J. Anim. Sci..

[73] Adams V.J., LeBlanc N., Penell J. (2022). Results of a Clinical Trial Showing Changes to the Faecal Microbiome in Racing Thoroughbreds after Feeding a Nutritional Supplement. Vet. Sci..

[74] Jacobasch G., Schmiedl D., Kruschewski M., Schmehl K. (1999). Dietary resistant starch and chronic inflammatory bowel diseases. Int. J. Color. Dis..

[75] Scheppach W., Sommer H., Kirchner T., Paganelli G.M., Bartram P., Christl S., Richter F., Dusel G., Kasper H. (1992). Effect of butyrate enemas on the colonic mucosa in distal ulcerative colitis. Gastroenterology.

[76] Hamer H.M., Jonkers D.M., Vanhoutvin S.A., Troost F.J., Rijkers G., de Bruine A., Bast A., Venema K., Brummer R.J. (2010). Effect of butyrate enemas on inflammation and antioxidant status in the colonic mucosa of patients with ulcerative colitis in remission. Clin. Nutr..

[77] Kovanda L., Hejna M., Du T., Liu Y. (2025). Butyrate Derivatives Exhibited Anti-Inflammatory Effects and Enhanced Intestinal Barrier Integrity in Porcine Cell Culture Models. Animals.

[78] Niederwerder M.C. (2018). Fecal microbiota transplantation as a tool to treat and reduce susceptibility to disease in animals. Vet. Immunol. Immunopathol..

